# Role of Complementary and Alternative Medicine in Controlling Dyslipidemia

**DOI:** 10.1155/2014/215731

**Published:** 2014-05-04

**Authors:** Waris Qidwai, Firdous Jahan, Kashmira Nanji

**Affiliations:** ^1^Family Medicine Department, Aga Khan University, Karachi 74800, Pakistan; ^2^Family Medicine Department, Oman Medical College, P.O. Box 391, Sohar, Oman

## 1. Introduction

This special issue focuses on the role of complementary and alternative medicine (CAM) in controlling dyslipidemia. Complementary and alternative medicine (CAM), also known as nonconventional medicine, includes a wide and heterogeneous array of health care practices (such as herbal medicine, acupuncture, yoga, meditation, and homeopathic medicine) that are not part of a health care system.

The popularity of CAM has dramatically increased in many developed countries since the 1990s. This could be attributed to the aging of population, prevalence of chronic diseases, and concern about the adverse reaction of chemical drugs. All these aspects have contributed greatly to the worldwide popularity of CAM. In the United States, consumers spend over $34 billion per year on CAM therapies spent outside the conventional health care financing system. This out-of-pocket expenditure is evidence of the belief that CAM therapies have benefits that outweigh their costs. CAM is generally more popular in most developed countries, especially in North America, Europe, and Australia. According to the 2007 National Health Interview Survey on more than 32,800 Americans, 38.2% of adults and 12% of children used some form of CAM within the previous 12 months.

Dyslipidemia is an independent preventable risk factor of coronary heart disease and has been shown to increase the risk of cardiovascular mortality manyfold. Therefore, the study on various indicators and risk factors of dyslipidemia appears to be significant for future health outcomes. It is critically important to recognize the need for treatment of dyslipidemia and to institute necessary therapies to reduce the long-term risks of disease recurrence or modify the metabolic derangements that promote atherosclerosis. Drugs used in dyslipidemia may cause adverse effects if used for longer duration. Therefore patients use CAM to reduce lipids without any major side effect.

The exact reasons for the popularity of CAM are complex; as they change with time and space, they vary from individual to individual and from therapy to therapy. In general, there are a broad range of positive motivations to the present popularity of CAM.

Although the use and expenditure of CAM have been increased radically, the potential role of CAM in modern clinical practice and health care system seems to be limited. As the efficacy and safety have been the major concerns in the recognition of CAM and integrating it into the conventional medicine, hence, there is an urgent need to provide evidence regarding the merits of the numerous techniques of CAM. The best way to achieve this is through rigorous research. Since then, in CAM, simple answers or broad generalizations are not possible. Each of the numerous techniques has to be evaluated separately. It is necessary to bring the practice of CAM in line with evidence. The wide dissemination of its findings is also important to include CAM into the modern clinical practice.

Studies have reported that CAM can be ineffective for a specific condition, but still they could be used as they do not harm the patients. Some argue that CAM should be used regardless of the results of placebo-controlled clinical trials, particularly when its use is not associated with serious risks.

The main purpose of this issue is to illustrate the inevitability of researches in the field of CAM to integrate it into the modern clinical practice. Altogether, 14 manuscripts were submitted for publication, out of which 11 manuscripts got accepted. The articles in this issue represent a wide range of therapeutic approaches of CAM in preventing dyslipidemia.

This special issue includes a review that discusses the progress and perspective of studies on dyslipidemia with single Chinese herb and its monomers or effective extracts during the past 10 years. The review concludes that traditional Chinese medicine (TCM) has some beneficial effects on the treatment of patients with dyslipidemia and has less adverse effects as compared to chemical agents. However, future clinical trials are needed to be confirmed about the effects of TCM.

This edition also includes a randomized, double-blind, placebo-controlled clinical trial on 50 hyperlipidemic patients. The results of this trial show that daily consumption of the fruit extract of* Vaccinium arctostaphylos* significantly reduces the serum levels of total cholesterol, LDL-C, and triglyceride (TG) and oxidative stress in hyperlipidemic patients. Therefore, this extract could be considered as a potential agent for treatment of dyslipidemia.

Another study in this issue is on Chaihu-Shugan-San (CSS), an ancient classical formula composed of seven Chinese herbs, which found that integrated recipe might work as a significant anti-inflammatory effect in Kupffer cells. In addition, another study conducted by Han et al. on the molecular docking of echinocystic acid (EA) (isolated from* G. sinensis* fruits) and the effects of it on the possible targets in* in vitro* rat liver microsomes has been added.

This review also includes a research paper which concluded that preventive acupuncture and moxibustion can significantly decrease the plasma TG and LDL, increase the plasma HDL, and prevent fat accumulation during climacteric period in rats. Moreover, a paper included is on intestinal transportations of main chemical compositions of Polygoni Multiflori Radix (PMR, originated from the root of* Polygonum multiflorum* Thunb.). This has been used in the treatment of hyperlipidemia in some countries for centuries. Another paper using the same herb PMR has been added to this special issue which discusses the mechanisms of the water extracts of Polygoni Multiflori Radix (PMR) and its processed products (PMRP) on liver lipid metabolism. Moreover, another paper was included in this review on propolis, which is a brownish resinous material collected by worker bees from the leaf buds of numerous plants that have strong pharmacological and biological properties.

Another study analyzed the effects of acupuncture on interleukin, IL-17expression in fat excess liver on adult mice, and provided some basic evidences that the inflammatory damage of hyperlipidemic fatty liver could be restricted through acupuncture. A study in this review investigates the beneficial effects of ethanol extract of* Gastrodia elata* Blume (EGB) on lipid metabolism and endothelial dysfunction in a high-fructose (HF) diet animal model.

## 2. Conclusion

The articles included in this issue highlight the need of sufficient scientific evidence from CAM research to clarify their mechanism of action and demonstrate their efficacy and safety. Patients and health care providers need to know which forms are safe and effective through unbiased scientific evaluation. Through the rigorous researches, the benefits of CAM therapies will be highlighted, and this will help in integration of CAM into the mainstream medicine. This integrative approach will ultimately lead to a safer and more effective patient-centered health care system.

